# The Estimated Prevalence of Hypospadias in Hokkaido, Japan

**DOI:** 10.2188/jea.14.73

**Published:** 2005-03-18

**Authors:** Norie Kurahashi, Masashi Murakumo, Hidehiro Kakizaki, Katsuya Nonomura, Tomohiko Koyanagi, Setsuko Kasai, Fumihiro Sata, Reiko Kishi

**Affiliations:** 1 Department of Public Health, Hokkaido University Graduate School of Medicine.; 2 Department of Renal and Genitourinary Surgery, Hokkaido University Graduate School of Medicine.

**Keywords:** hypospadias, prevalence, endocrine-disrupting chemicals, population-based registry systems

## Abstract

BACKGROUND: Hypospadias is one of the most common congenital anomalies in the world. Recently, increases in the prevalence of hypospadias have been reported in various countries including Japan. In this study, we examined whether the prevalence of hypospadias in Hokkaido, Japan, increased or not, using standardized diagnostic criteria. We also investigated the degree of its severity.

METHODS: We calculated prevalence of hypospadias using hospital records of hypospadias repair in Hokkaido. The prevalence from 1985 through 1997 by dividing the number of patients obtained from hospital records by the number of births.

RESULTS: The average prevalence of hypospadias in Hokkaido was 3.9 per 10,000 births, and did not significantly changed (p=0.7). The average proportions of distal, proximal and chordee alone were 56.7%, 39.6% and 3.7%, respectively. The decrease in the proportion of the proximal type was statistically significant (p=0.05) for the entire time period, whereas the proportion of the distal type did not have a significant upward trend for the observed 13 years (p=0.1).

CONCLUSION: No significant changes in the prevalence of hypospadias existed in Hokkaido.

Hypospadias is incomplete fusion of the urethral folds resulting in a urethral opening on the ventral surface of the penis or on the scrotum, or perineum. The prevalence of hypospadias varies widely in different countries and populations, ranging from 0.37 to 41 per 10,000 infants.^1^

The etiology of hypospadias is still unknown. Because male sexual differentiation is critically dependent on normal androgen concentrations, increased exposure to environmental factors affecting androgen homeostasis during fetal life (i.e. endocrine-disrupting chemicals with estrogenic or anti-androgenic effects) may cause hypospadias.^2^^-^^5^

According to the International Clearinghouse for Birth Defects Monitoring System, an increase in the prevalence of hypospadias has been reported in various countries, including the United States of America, Norway, Sweden, the United Kingdom, Hungary, Denmark, Italy, France, and Japan.^6^ In Japan, nationwide hospital-based monitoring started in 1972 and the Japan Association of Obstetricians and Gynecologists has been a member of the International Clearinghouse for Birth Defects Monitoring System since 1988. The prevalence of hypospadias in Japan in 1974, 1985 and 1998 was 1.4, 2.5, and 3.5 per 10,000 births, respectively. Thus, an increasing trend in the prevalence of hypospadias in Japan was observed.^7^ On the other hand, there are some population-based monitoring systems in Japan, such as those in Kanagawa, Tottori, and Ishikawa. The prevalence of hypospadias in Kanagawa in 1981-83, 1984-86, 1989-93, and 1994-94 was 3.9, 4.6, 5.1, and 2.9 per 10,000 births, respectively. In Ishikawa in 1983-87, 1998-92, and 1993-97, it was 1.4, 3.8, 3.0, and 2.5 per 10,000 male births, respectively. In Tottori in 1974-81, 1982-89, and 1990-96, the prevalence was 1.8, 5.3, and 6.3 per 10,000 births, respectively, but this system changed the method of registry in each period.^8^ According to another hospital-based monitoring survey in Osaka, Japan, the prevalence of hypospadias was 1.39 per 10,000 male births during 1948-1958, but 12.13 during 1981-1990; that is, it increased 9.7 fold.^9^

It is difficult to compare reported rates because of differences in diagnostic criteria (inclusion or exclusion of minor cases). Moreover, physicians’ criteria for referral of boys with hypospadias may have changed over time with the widespread recognition that even the mild form of hypospadias should be treated surgically. Thus, the reported number of mild forms of hypospadias may be increasing.^10^ On the other hand, Paulozzi, et al. recently reported that the rate of severe cases increased in the United State,^11^ but there has been no report about the rates of severity of hypospadias in Japan.

In this study, using standardized criteria,^12^ we examined whether the prevalence of hypospadias in Hokkaido, Japan, increased. We also investigated the severity of the disease.

## METHODS

We calculated the prevalence of hypospadias using hospital records of operations for hypospadias. We obtained all the information in 2000. We identified patients who were surgically operated on for hypospadias from 1985 through 2000 in 12 major hospitals in Hokkaido that had surgeons who could perform such operations (Hokkaido University Medical Hospital, Sapporo Medical University Hospital, Asahikawa Medical College Hospital, Sapporo Municipal Hospital, Asahikawa Municipal Hospital, Asahikawa Kosei General Hospital, Obihiro Kosei General Hospital, Hakodate Chuo General Hospital, Muroran Municipal Hospital, Kushiro Municipal Hospital, Kushiro Rosai Hospital, and Iwamizawa Municipal Hospital). In these 12 major hospitals, we were able to count all of the hypospadias cases requiring operation. We excluded patients born outside Hokkaido.

We collected information on the patients such as the date of birth, the year of operation and the severity of hypospadias from the hospital records. Hypospadias is classified as distal when the opening of the urethra is in the penile, coronal or glandular portion, and as proximal when the opening of the urethra is in the penoscrotal, scrotal or perineal portion. The urologists who operated on the patients decided the degree of hypospadias.

We obtained the number of births (males and females) from the annual statistical report on health in Hokkaido.^13^ Then we estimated the prevalence of hypospadias by dividing the number of patients by the number of births in Hokkaido. The prevalence of hypospadias is presented as the number of cases per 10,000 births.

The average age at operation between 1985 and 2000 was 3.03 years (standard deviation: 1.6 years). We estimated the trend of the hypospadias prevalence from 1985 through 1997 because we assumed that the patients who were born in and after 1998 had not been operated on yet.

Analysis of the trend over time of the prevalence was based upon simple linear regression. Statistical significance was determined by p<0.05. The statistical package SPSS^®^ 10.1 was used for these analyses.

## RESULTS

**Figure 1.**
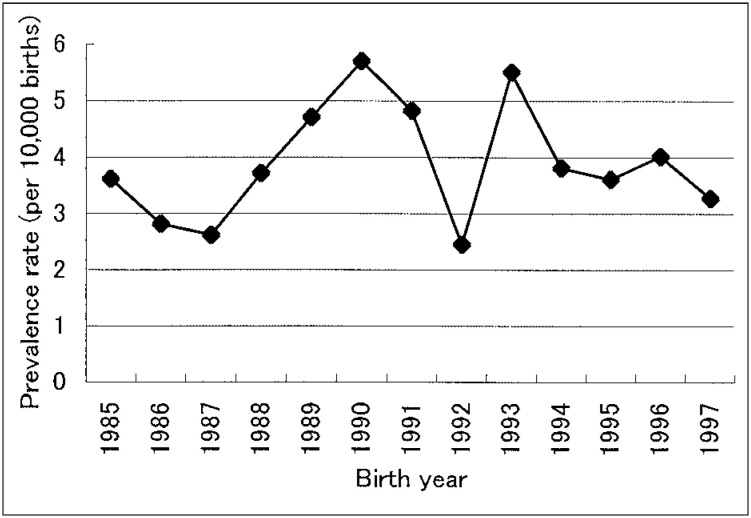
Prevalence of hypospadias by calendar year in Hokkaido.

A total of 278 hypospadias cases were reported from the 12 major hospitals in Hokkaido. Overall, the average prevalence of hypospadias for 13 years was 3.9 per 10,000 births (Figure 1). The highest rate was recorded in 1990 (5.7 per 10,000 births) and the lowest in 1992 (2.4 per 10,000 births). There was no increasing or decreasing tendency. The prevalence between 1985 and 1990 demonstrates on upward trend, but it was not significant (p=0.06), and a downward trend existed between 1991 and 1997 but it was also without statistical significance (p=0.6).

**Figure 2.**
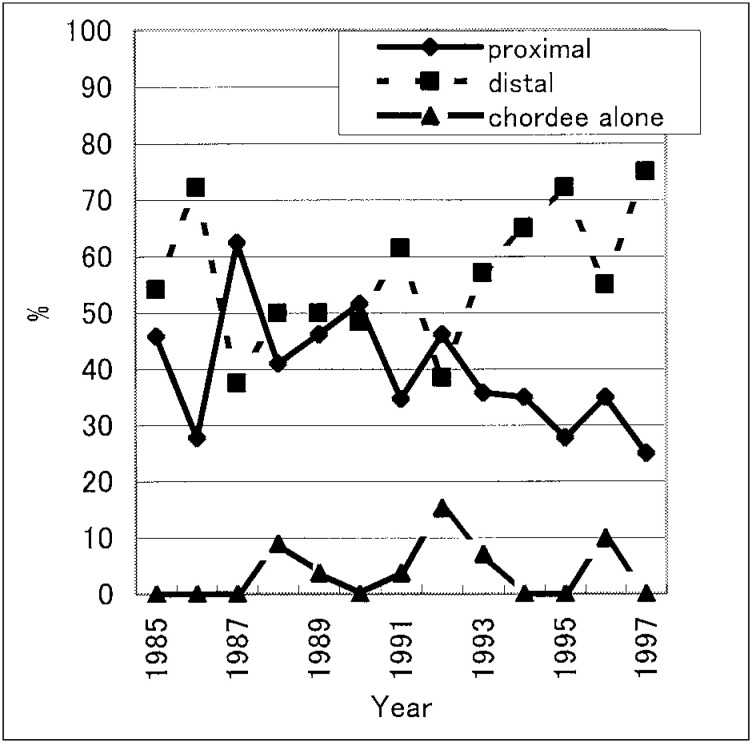
Proportion of hypospadias classified by the degree of severity in Hokkaido.

**Figure 3.**
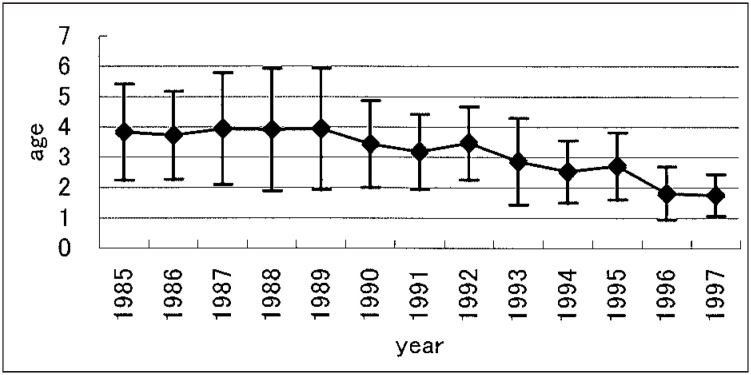
Average and standard deviation of age at surgical operation for hypospadias in Hokkaido.

Concerning the severity of hypospadias, average proportions of distal, proximal and chordee alone were 56.7%, 39.6% and 3.7%, respectively. The decrease in the proportion of the proximal type was statistically significant (p=0.05) for the entire time period, whereas the proportion of the distal type for 13 years did not demonstrate any significant upward trend (p=0.1). The proportion of chordee alone did not show any significant increase or decrease (p=0.5). (Figure 2). The average of age at operation significantly fell with time (p<0.0001) (Figure 3), and ranged from 1 to 10 years (mean: 3.03 years).

## DISCUSSION

We collected the clinical records of the patients with hypospadias in all the 12 hospitals where urologists were able to operate for hypospadias between 1985 and 2000 in Hokkaido. The urologists in Hokkaido University have worked on improving hypospadias surgery via techniques such as One stage Urethroplasty with Parameatal Flap,^14^^,^^15^ and experienced many cases of hypospadias. Therefore, most patients with hypospadias in Hokkaido have attended the major leading hospitals such as Hokkaido University Medical Hospital. Moreover, in Hokkaido, all children are examined by pediatricians at least twice during their first 1 year of life; even if children with hypospadias are missed at birth, therefore, all of them are referred to a specialist for evaluation and treatment. Furthermore, our policy is to treat all cases of hypospadias surgically, even mild hypospadias. Additionally, the rate of moving to other prefectures from Hokkaido is the smallest in the country.^16^ Thus, it seems that few patients with particular congenital malformations such as hypospadias have migrated to other prefectures. Thus, we expected to count all the cases with hypospadias in Hokkaido except for cases who moved to other prefectures or cases with severe congenital heart disease who could not receive surgery for hypospadias. Therefore, this may be the best way to estimate the prevalence of hypospadias in Hokkaido because there are no population-based registry systems of congenital malformations in Japan.

Moreover, as far as we know, there have been few epidemiologic reports on the degree of severity of hypospadias in Japan. The diagnostic criteria have not been perfectly defined. However, recently a new method of classification was proposed; the severity of hypospadias classified into glanular, distal and proximal types.^12^ Our classification in this study is almost same, except for including glanular together in distal. The diagnostic criteria for hypospadias were established in Hokkaido University early in the 1980s and have spread widely all over Hokkaido since then. We did not make any changes in the criteria or in the treatment policy that might affect the results of the current study. Therefore, to examine the prevalence of hypospadias and the data about proportions of the degree of hypospadias in Hokkaido are worthwhile and will provide a good reference for future studies.

In the present study, the estimated average prevalence of hypospadias between 1985 and 1997 was 3.9 per 10,000 live births, and the prevalence in Hokkaido did not show any increasing or decreasing tendency. Neither the prevalence in Kanagawa nor that in Ishikawa showed any increasing or decreasing tendency as well.^8^ On the other hand, there was an increasing tendency in the Japan Association of Obstetricians and Gynecologists^8^ study and in Osaka^9^. However, it is difficult to compare the rates previously reported because of differences in diagnostic criteria. For example, according to the birth defect definition of the International Clearinghouse for Birth Defects Monitoring System (the Japan Association of Obstetricians and Gynecologists is a member of the International Clearinghouse for Birth Defects Monitoring System), glandular hypospadias is excluded. This is one of the reasons why the prevalence in Hokkaido was higher than in the Japan Association of Obstetricians and Gynecologists study. In 1998, the prevalence of hypospadias ascertained by the Japan Association of Obstetricians and Gynecologists was 3.5 per 10,000 births, which is very similar to our results, but higher than the rates reported for earlier years. This apparent increase, however, may be explained by improved registration and a change in diagnostics. Concerning Tottori, the method was changed from hospital-based to population-based during the observational period.^8^

In comparison with other countries, our reported rate was lower than those in the Netherlands (38 per 10,000 births),^17^ Finland (28.1 per 10,000 male births),^10^ and the United State (39.7 per 10,000 births).^11^ These results suggest that there are also geographical differences in the prevalence of hypospadias and that the etiology of hypospadias involves not only endocrine-disrupting chemicals but also genetic and racial factors.

There are a few reports suggesting a difference of prevalence between mild and severe degrees of hypospadias. In the United States, Paulozzi, et al. reported that the rate of severe hypospadias increased between 1968 and 1993.^11^ In contrast, our study showed a significant decrease in the proportion of the severe (proximal) type between 1985 and 1997. In our study, hypospadias was classified as distal when the opening of the urethra was in the penile, coronal, or glandular portion and as proximal when the opening of the urethra was in at penoscrotal, scrotal, or perineal portion. On the other hand, Paulozzi, et al. classified it as mild when the urethral opening was on the ventral surface of the glans penis and as severe when the opening was on the ventral surface of the shaft, or was scrotal or perineal.^11^ Thus, it is difficult to compare our data with previous data because of the differential criteria.

The etiology of hypospadias has been hypothesized to be a disturbance of endogenous hormonal balance, or exposure to the exogenous endocrine-disrupting chemicals because the development of the male external genital organs is under hormonal control. In an animal experiment, hypospadias was induced by in utero exposure to phthalate ester, which has anti-androgen effects.^18^ In an epidemiologic study, Klip, et al. suggested that the risk of hypospadias increased in the sons of pregnant women exposed to diethylstilbestrol.^19^ Sakakibara, et al. reported a possible link between hypospadias and in utero exposure to progesterone, which was used for treatment of spontaneous abortion.^20^ Moreover, Kristensen, et al. reported that hypospadias was associated with exposure to pesticides.^21^ Thus, these investigations have focused on the association between hypospadias and endocrine-disrupting chemicals with estrogenic or anti-androgenic effects. There is another report suggesting a relation between the area of the penis and endocrine-disrupting chemicals. Kalloo, et al. reported that the stroma of the glans was androgen receptor-rich, but that the epithelia of the penile shaft skin and scrotal skin were androgen receptor negative in human male fetuses at 18-22 weeks gestation.^22^ Thus, it is possible that the trend of hypospadiac severity might reflect sensitivity to the endocrine-disrupting chemicals. Therefore, it is important that we keep monitoring the trend of hypospadiac severity.

Our study indicated that the age at operation had become significantly younger. This was caused by an improved understanding of the psychological implications of genital surgery in children, improvements in the technical aspects of surgery for hypospadias and advances in pediatric anesthesia.^23^

There are a few limitations in the present study. First, we could not include the cases who were not operated on because of severe co morbidities such as congenital heart disease. Second, we excluded patients who were not born in Hokkaido and we could not count the patients who might have been operated on outside Hokkaido. Therefore, the true prevalence might be a little higher or this study may have underestimated it. This might be another reason why the prevalence of hypospadias in Hokkaido is lower than in Osaka.

To prevent hypospadias, considering the relation with environmental factors, we need to find risk factors, but we did not find any in this study because we collected data from hospital records. We could not obtain information such as maternal age at delivery, the place of birth, familial history and environmental factors. Fisch, et al. reported that the prevalence of hypospadias did not change between 1983 and 1996, but that a risk factor of hypospadias was high maternal age at delivery.^24^ In Finland, an association between the prevalence of hypospadias and remoteness from the closest city was observed.^25^ It is necessary to find risk factors to prevent hypospadias in future studies.

This study did not find any significant change in the prevalence of hypospadias, but proximal hypospadias significantly decreased in Hokkaido. Because there are different registries, ages at diagnosis, and diagnostic criteria around the world, it is difficult to conclude that the prevalence of hypospadias is increasing worldwide. Therefore, it is important to accumulate data for prevention of the disease in Japan. In the future, to monitor hypospadias rates and trends accurately, we hope to establish a population-based registry system in Japan.

## References

[1] Kallen B, Bertrollini R, Castilla E, Czeizel A, Knudsen LB, Martinez-Frias ML, . A joint international study on the epidemiology of hypospadias. Acta Paediatr Scand 1986;324(Suppl):1-52. 10.1111/j.1651-2227.1986.tb14935.x 3471045

[2] Sharpe RM, Skakkebaek NE. Are oestrogens involved in falling sperm counts and disorders of the male reproductive tract? Lancet 1993;341:1392-5. 8098802 10.1016/0140-6736(93)90953-e

[3] Sharpe RM. Hormones and testis development and the possible adverse effects of environmental chemicals. Toxicol Lett 2001;120:221-32. 11323180 10.1016/s0378-4274(01)00298-3

[4] Skakkebaek NE, Rajpert-De Meyts E, Main KM. Testicular dysgenesis syndrome: an increasingly common developmental disorder with environmental aspects. Hum Reprod 2001;16:972-8. 11331648 10.1093/humrep/16.5.972

[5] Sultan C, Balaguer P, Terouanne B, Georget V, Paris F, Jeandel C, . Environmental xenoestrogens, antiandrogens and disorders of male sexual differentiation. Mol Cell Endocrinol 2001;178:99-105. 11403899 10.1016/s0303-7207(01)00430-0

[6] Paulozzi LJ. International trends in rates of hypospadias and cryptorchidism. Environ Health Perspect 1999;107:297-302. 10090709 10.1289/ehp.99107297 PMC1566511

[7] Sumiyoshi Y, Hirahara F, Sakamoto S. Studies on the frequency of congenital malformations in Japan and Asian countries. Congenit Anom 2000;40:S76-86.

[8] Sumiyoshi Y, Hirahara F, Asakura H, Sakamoto S, Takeshita K, Nakagawa H, . Report of congenital anomalies in Japan. Sanfujinka-no-Sekai (in Japanese). 2000;53:737-48.

[9] Imaizumi Y, Yamamura H, Nishikawa M, Matsuoka M, Moriyama I. The prevalence at birth of congenital malformations at a maternity hospital in Osaka City, 1948-1990. Jinrui Idengaku Zassi 1991;36:275-87. 10.1007/BF01910546 1753441

[10] Aho M, Koivisto AM, Tammela TL, Auvinen A. Is the incidence of hypospadias increasing? Analysis of Finnish hospital discharge data 1970-1994. Environ Health Perspect 2000;108:463-5. 10.1289/ehp.00108463 PMC1638043 10811575

[11] Paulozzi LJ, Erickson JD, Jackson RJ. Hypospadias trends in two US surveillance systems. Pediatrics 1997;100:831-4. 9346983 10.1542/peds.100.5.831

[12] Hadidi AT. Classification of hypospadias. Hadidi AT and Azmy AF, eds. Hypospadias surgery: An illustrated guide. Springer-Verlag Berlin Heidlberg. 2004.

[13] Hokkaido Department of Health and Welfare. Annual Report of Health Statistics in Hokkaido. Hokkaido Department of Health and Welfare. Sapporo, 2000.

[14] Koyanagi T, Matsuno T, Nonomura K, Sakakibara N. Complete repair of severe penoscrotal hypospadias in 1 stage: experience with urethral mobilization, with flap-flipping urethroplasty and “glanulomeatoplasty”. J Urol 1983;130:1150-4. 6644897 10.1016/s0022-5347(17)51732-2

[15] Koyanagi T, Nonomura K, Gotoh T, Nakanishi S, Kakizaki H. One-stage repair of perineal hypospadias and scrotal transposition. Eur Urol 1984;10:364-7. 6529948 10.1159/000463834

[16] Bureau of Statistics Office of the Prime. Annual report on the internal migration in Japan derived from the basic resident registers. Japan Statistical Association.2002.

[17] Pierik FH, Burdorf A, Nijman JM, de Muinck Keizer-Schrama SM, Juttmann RE, Weber RF. A high hypospadias rate in The Netherlands. Hum Reprod 2002;17:1112-5. 11925415 10.1093/humrep/17.4.1112

[18] Gray LE Jr., Ostby J, Furr J, Price M, Veeramachaneni DN, Parks L. Perinatal exposure to the phthalates DEHP, BBP, and DINP, but not DEP, DMP, or DOTP, alters sexual differentiation of the male rat. Toxicol Sci 2000;58:350-65. 11099647 10.1093/toxsci/58.2.350

[19] Klip H, Verloop J, van Gool JD, Koster ME, Burger CW, van Leeuwen FE. Hypospadias in sons of women exposed to diethylstilbestrol in utero: a cohort study. Lancet 2002;359:1102-7. 11943257 10.1016/S0140-6736(02)08152-7

[20] Sakakibara N, Nonomura K, Matsuno T, Koyanagi T. Some clinical and endocrinological study of hypospadias. Nippon Hinyokika Gakkai Zassi 1985;76: 716-22. (in Japanese) 10.5980/jpnjurol1928.76.5_716 3932737

[21] Kristensen P, Irgens LM, Andersen A, Bye AS, Sundheim L. Birth defects among offspring of Norwegian farmers, 1967-1991. Epidemiology 1997;8:537-44. 9270956 10.1097/00001648-199709000-00011

[22] Kalloo NB, Gearhart JP, Barrack ER. Sexually dimorphic expression of estrogen receptors, but not of androgen receptors in human fetal external genitalia. J Clin Endcrinol Metab 1993;77:692-8. 10.1210/jcem.77.3.8370691 8370691

[23] American Academy of Pediatrics Section on Urology. Timing of elective surgery on the genitalia of male children with particular reference to the risks, benefits, and psychological effects of surgery and anesthesia. Pediatrics 1996;97:590-4. 8632952

[24] Fisch H, Golden RJ, Libersen GL, Hyun GS, Madsen P, New MI, . Maternal age as a risk factor for hypospadias. J Urol 2001;165:934-6. 11176518

[25] Aho MO, Koivisto AM, Tammela TL, Auvinen AP. Geographical differences in the prevalence of hypospadias in Finland. Environ Res 2003;92:118-23. 12854691 10.1016/s0013-9351(02)00089-0

